# Deep learning for classifying the stages of periodontitis on dental images: a systematic review and meta-analysis

**DOI:** 10.1186/s12903-023-03751-z

**Published:** 2023-12-19

**Authors:** Xin Li, Dan Zhao, Jinxuan Xie, Hao Wen, Chunhua Liu, Yajie Li, Wenbin Li, Songlin Wang

**Affiliations:** 1 School of Public Health, National Institute for Data Science in Health and Medicine, Capital Medical University, Beijing, China; 2 Department of Implant Dentistry, Beijing Stomatological Hospital, Capital Medical University, Beijing, China; 3 City University of Hong Kong, Hong Kong SAR, China; 4 Beijing Tiantan Hospital, Capital Medical University, Beijing, China; 5 Salivary Gland Disease Center and Beijing Key Laboratory of Tooth Regeneration and Function Reconstruction, Beijing Laboratory of Oral Health and Beijing Stomatological Hospital, Capital Medical University, Beijing, 100050 China

**Keywords:** Periodontitis, Deep learning, Convolutional neural networks, Dental images

## Abstract

**Background:**

The development of deep learning (DL) algorithms for use in dentistry is an emerging trend. Periodontitis is one of the most prevalent oral diseases, which has a notable impact on the life quality of patients. Therefore, it is crucial to classify periodontitis accurately and efficiently. This systematic review aimed to identify the application of DL for the classification of periodontitis and assess the accuracy of this approach.

**Methods:**

A literature search up to November 2023 was implemented through EMBASE, PubMed, Web of Science, Scopus, and Google Scholar databases. Inclusion and exclusion criteria were used to screen eligible studies, and the quality of the studies was evaluated by the Grading of Recommendations Assessment, Development and Evaluation (GRADE) methodology with the QUADAS-2 (Quality Assessment of Diagnostic Accuracy Studies) tool. Random-effects inverse-variance model was used to perform the meta-analysis of a diagnostic test, with which pooled sensitivity, specificity, positive likelihood ratio (LR), negative LR, and diagnostic odds ratio (DOR) were calculated, and a summary receiver operating characteristic (SROC) plot was constructed.

**Results:**

Thirteen studies were included in the meta-analysis. After excluding an outlier, the pooled sensitivity, specificity, positive LR, negative LR and DOR were 0.88 (*95%CI* 0.82–0.92), 0.82 (*95%CI* 0.72–0.89), 4.9 (*95%CI* 3.2–7.5), 0.15 (*95%CI* 0.10–0.22) and 33 (*95%CI* 19–59), respectively. The area under the SROC was 0.92 (*95%CI* 0.89–0.94).

**Conclusions:**

The accuracy of DL-based classification of periodontitis is high, and this approach could be employed in the future to reduce the workload of dental professionals and enhance the consistency of classification.

**Supplementary Information:**

The online version contains supplementary material available at 10.1186/s12903-023-03751-z.

## Background

Since the 1990s, periodontitis has been a global public health burden, and severe periodontitis, with a 10.59% prevalence rate, ranks 6th among 369 assessed diseases and is responsible for 7.09 million disability-adjusted life years (DALYs), according to the 2019 Global Burden of Diseases (GBD) study [1–3]. Periodontitis affects local health and systemic conditions, meaning that if periodontitis is properly treated, systematic inflammation will be reduced [4–8]. However, manual classification based on dental images requires a lot of manpower and time. Furthermore, image quality and radiographic interpretation could compromise the accuracy of classification. All these issues could be alleviated by deep learning (DL) methods [9–11].

Both DL and machine learning (ML) are included in artificial intelligence (AI). ML aims at self-training algorithms based on existing data and making predictions for new information [12]. DL is a subgroup of ML that mimics the way the human brain works and is based on neural network structures [13]. Recently, DL, especially convolutional neural networks (CNNs), has been widely used in various fields of medical image analysis, such as segmentation, detection, classification of abnormality, and computer-aided diagnosis [14]. CNNs identify visual patterns directly from the raw pixels of an image, which is similar to the way humans observe objects, to learn the intrinsic features or patterns of the image [14]. They are multi-layered, feed-forward, neural networks using backpropagation algorithms, and consist of convolutional, activation, and pooling layers. Currently, CNNs are still considered the most successful method to process medical images [15].

In dentistry, there are four main applications of CNNs: (1) segmentation; (2) detection; (3) classification; and (4) image quality enhancement, which are all based on dental images, including intraoral (periapical radiograph and bite-wing image) and extra-oral (panoramic X-ray and cone-beam computed tomography [CBCT]) X-rays [9, 16]. For instance, Park et al. applied CNNs to segment tooth surfaces for caries diagnosis [17], and Lee et al. proposed a computer-assisted detection system to identify impacted mandibular third molar teeth [18]. Nowadays, there is a growing trend in the utilization of CNNs in periodontitis fields. Jaiswal et al. developed a novel Intelligent Ant Lion-based Convolution Neural Model (IALCNM) to segment affected parts and classify the wear and periodontitis using panoramic photographs [19]. Moreover, Chen et al. developed an ensembled CNN model to predict tooth position and recognize radiographic bone loss (RBL) using periapical and bitewing radiographs [20]. Furthermore, Moran et al. evaluated whether different pre-processing methods affect the result of periodontal bone loss (PBL) classification based on periapical images [21].

Although there are numerous studies conducted in the interdisciplinary of periodontitis and DL, the type of DL architecture employed in periodontitis classification, determination of the most effective model and comparison of performance against oral physicians have not been systematically reported. Therefore, this study aimed to review the studies on the classification of periodontitis by evaluating various dental images using DL methods, to summarise the types of different models employed, and to compare the performance of these models. This could identify the most appropriate model for the classification of periodontitis based on oral photographs in clinical practice. Moreover, we compared the performance of the DL model to the dental professionals which determines the reliability.

## Methods

This systematic review and meta-analysis were conducted referring to the guidelines for Preferred Reporting Items for Systematic Reviews and Meta-analyses for Diagnostic Test Accuracy Studies (PRISMA-DTA). The study was registered at the National Institute for Health Research, International Prospective Register of Systematic Reviews (PROSPERO, registration number CRD 42022338627). Additionally, the study protocol was based on the following PIRD elements [22]:

### Population

patients’ diagnostic images that illustrate the status of radiographic bone loss (RBL).

### Index test

deep learning models for classification of periodontitis based on RBL.

### Reference test

expert opinions according to the classification of periodontitis.

### Diagnosis of interest

classification of periodontitis.

### Data sources

A reviewer (XL) searched publications through EMBASE, PubMed, Web of Science, Scopus and Google Scholar databases up to November 2023 according to strategies set by two reviewers (DZ and XL). Search strategies combined terms including (1) periodontitis or periodontal disease or periodontal status; (2) image or image processing or computer-aided diagnosis or computer-based diagnosis or smart diagnosis; and (3) artificial intelligence or machine learning or deep learning or convolutional neural networks. The detailed search queries for all databases were provided in Supplementary Table 1.

### Criteria for considering studies for this review

Studies that matched the following criteria were considered to be included: (1) Study population with a dental image; (2) Diagnosing with DL technology; and (3) English publications with all statuses, including in-press and unpublished studies. The exclusion criteria were: (1) Animal experiment; (2) Without full article; (3) Without statistical data; and (4) Conference proceedings or reviews or books or patents. (Table 1)

**Table 1:** Inclusion and exclusion criteria for this review

Inclusion criteria	Study population with a dental image
	Diagnosing with DL technology
	English publications with all statuses, including in-press and unpublished studies
Exclusion criteria	Animal experiment
	Without full article
	Without statistical data
	Conference proceedings or reviews or books or patents

### Study selection and data collection

After screening the titles and abstracts of all identified publications, two reviewers (XL and JXX) independently read the full text of all eligible articles and excluded inappropriate articles according to the inclusion/exclusion criteria. Disagreements between the reviewers were solved by discussing until a consensus was reached or by consulting a third reviewer (DZ). The following data were extracted from each publication: study characteristics (first author, publication year, country), study design (data sets, modality of medical images, machine learning algorithms, study factor, and its definition, algorithms application, comparison), primary outcomes, and conclusions.

### Quality assessment

The quality of evidence was evaluated by the Grading of Recommendations Assessment, Development and Evaluation (GRADE) on the following domains: study design, limitations (risk of bias), indirectness, inconsistency, imprecision, and publication bias (https://gdt.gradepro.org/) [23]. The quality of evidence was categorized into four levels: high, moderate, low and very low.

Based on the recommendation of the Cochrane Collaboration, the QUADAS-2 (Quality Assessment of Diagnostic Accuracy Studies) tool was used to evaluate the quality of all eligible articles in terms of the risk of bias and applicability [24]. The assessment was conducted by three reviewers (XL, JXX and YJL). When there were disagreements, it was resolved by discussion or by consulting a third reviewer (DZ) to make the final decision. There were four domains for the risk of bias section: patient selection, index test, reference standard, and flow and timing; the first three of these domains formed the applicability section [25].

### Statistical analysis

Summarising the quality score to define high-quality studies is not a recommended method [26]. Moreover, the overall estimate may be similar regardless of the quality of the studies, but if only high-quality studies are analyzed, incomplete reporting may arise [27]. Therefore, all articles containing true positive (TP), false positive (FP), true negative (TN) and false negative (FN) data that were either supplied in the articles or could be calculated from the information provided were used to conduct a meta-analysis using Stata 16.0 software (StataCorp LLC, College Station, TX, USA). Spearman correlation analysis was conducted to assess the threshold effect, without which combined sensitivity, specificity, positive likelihood ratio (LR), negative LR and diagnostic odds ratio (DOR) were calculated directly by using the random-effects inverse-variance model. A forest plot of sensitivity and specificity was generated to visually show the differences among the included studies. Statistical heterogeneity was assessed using the Chi-squared–based Q statistic method and *I*^*2*^, and the level of significance was indicated by *P* < 0.05 and *I*^*2*^ > 50%, respectively. Influence analysis and subgroup analysis based on study factors including article quality (high/unclear risk of bias, low risk of bias), dental image modality (periapical radiograph images, panoramic dental radiographs), model type (single model, two-stage model) were performed to detect the source of heterogeneity. Two meta-regression models with sensitivity and specificity were carried out to investigate whether sample size has an impact on classification outcomes. A summary receiver operating characteristic (SROC) plot—a plot of scattered sensitivity-specificity points of each potentially eligible study—was constructed, and the area under SROC (AUSROC) was computed [24]. In addition, a Fagan nomogram was drawn to describe how DL methods may have helped clinicians increase the probability of an effective classification of periodontitis. Publication bias was investigated by Deeks’ funnel plot asymmetry test.

## Results

### Study selection

**Fig. 1:**
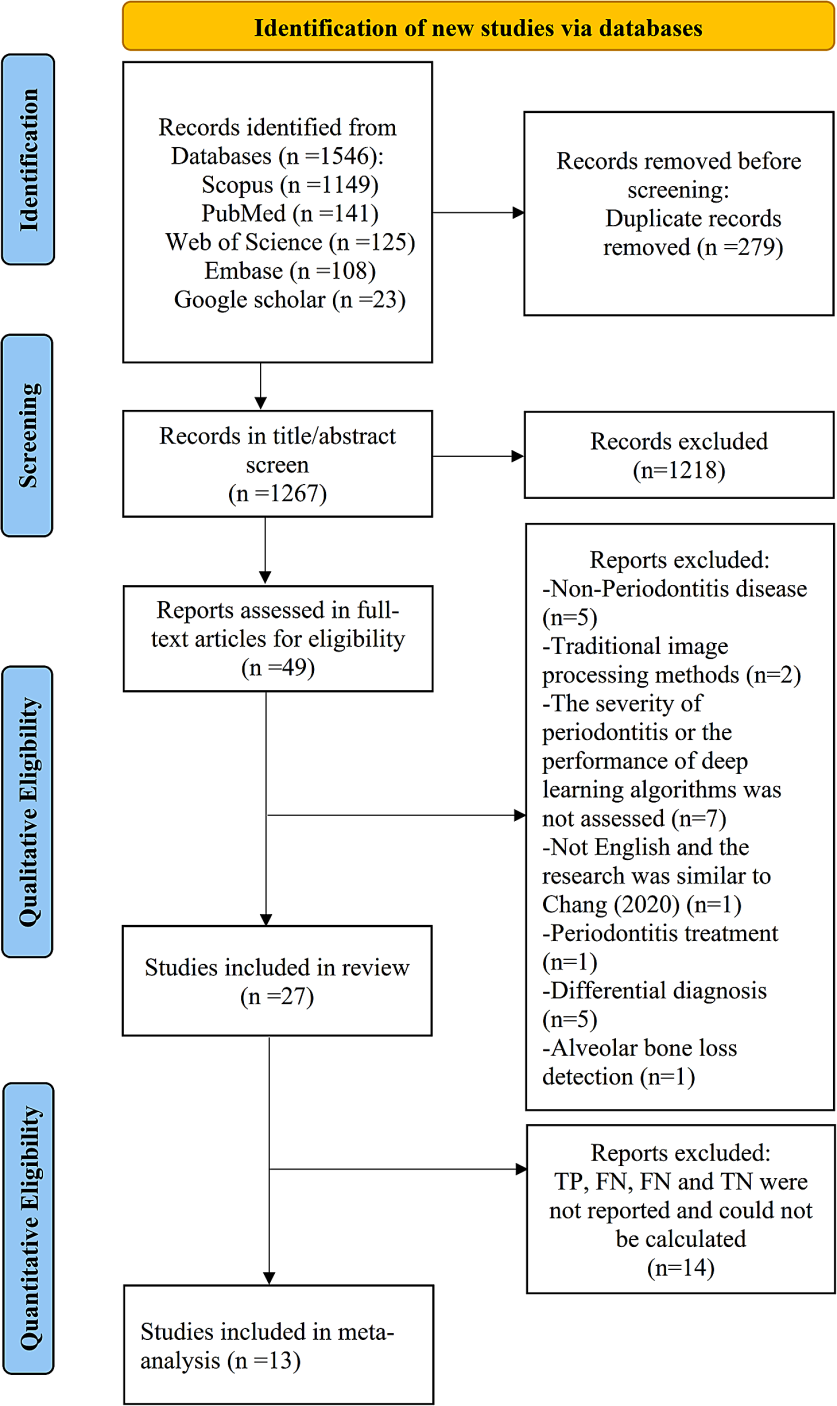
PRISMA Flow chat of study selection process

Figure 1 shows the study selection process and describes the reasons for full-text article exclusion. The five databases (EMBASE, PubMed, Web of Science, Scopus and Google Scholar) identified 1546 potentially relevant publications with 279 duplications. After screening the titles and abstracts of the 1267 remaining studies, 49 articles were selected for full-text reading. Based on the inclusion and exclusion criteria, 27 studies were included in this systematic review [20, 21, 28–52].

### Methodological quality

The risk of bias and applicability were assessed using QUADAS-2 for all included articles, and the results were shown in Supplementary Fig. 1 and Supplementary Fig. 2, respectively. Nearly half of the included studies did not have clear information on whether patients were consecutively or randomly enrolled, resulting in 42.9% of the articles (12/27) showing an unclear risk of bias in the patient selection domain [20, 30, 32, 34–36, 38, 45, 48, 52, 37, 42]. Two studies were rated as having a high risk of bias, with one [29] designed to be a case-control study with a convenient sample collection and the other [31] using inappropriate exclusion criteria. Approximately one-fourth of the studies did not mention a prespecified threshold before a test, consequently, 22.2% of the articles (6/27) were ranked as having unclear risk of bias in the index test domain [21, 35, 39, 49, 51, 52]. Four studies were unable to accurately diagnose periodontitis based on their reference tests, as these studies attempted to classify healthy cases and periodontitis only using radiographs [21, 28, 42, 49]. The other studies (85.2%, 23/27) were ranked as having a low risk of bias in the reference standard domain [20, 29–41, 43–48, 50–52]. As the diagnostic tests are being conducted by DL algorithms, which do not affect the flow and timing, all articles in the present analysis were ranked as low risk. For the applicability section, all studies were ranked at low risk of bias in patient selection, 74.1% of the included studies (20/27) were ranked as low risk of bias in the index test and reference standard [20, 29, 30, 32–34, 36–48, 52]. The study quality assessment results are presented in Supplementary Table 2.

The quality of evidence based on the GRADE analysis can be found in Supplementary Table 3. Results are shown in different subgroups of model type and dental image modality. When one study was ranked as high risk of bias or unclear risk of bias based on QUADAS-2, the subgroup’s limitation was assessed as a high risk of bias. As a result, all subgroups were considered to be at high risk of bias, leading to one level of evidence quality deduction. Two level of evidence quality was downgraded in the single model using periapical radiograph images and two-stage model subgroups due to inconsistency and imprecise data. While one level of evidence quality was reduced in the single model using panoramic dental radiographs. Consequently, the quality of evidence was scored as very low in the single model with periapical radiograph images and the two-stage model and low in the single model with panoramic dental radiograph.

### Study characteristics

The characteristics of all included studies are summarised in Table 2. All articles were published within the last five years, and there was a surge in 2021 with twice as many articles published than in 2020, while in 2022, the number of articles published was 1.5 times that of 2021 (Supplementary Fig. 3). Studies originated from 11 countries, most of which were in Asia. Except for one study that never mentioned data splitting [20], all included studies (26/27) split the datasets or used cross-validation, an approach to avoid model overfitting and evaluate the generalization ability of the model. Three studies used an external dataset to evaluate the performance of the algorithms [29, 43, 48]. In addition, three studies used public databases [35–37]. In terms of dental image modality, the studies employed periapical radiograph images, panoramic dental radiographs, and CBCT images to classify periodontitis, among which panoramic radiographs were used the most (15/27) [28–30, 32, 33, 35, 36, 38, 39, 42, 47–51] and only one study used CBCT [44]. More than two-thirds of articles (19/27) processed images before applying DL techniques by some common approaches, such as augmentation, normalisation and resizing the images [21, 28, 29, 31–34, 36, 38–40, 43–45, 47, 48, 50–52]. Furthermore, the DL-aided task has changed over time. In 2019 and 2020, the diagnosis of periodontitis was predominantly chosen, whereas the classification of periodontitis stages was selected in 2021 and 2022. Half studies opted diagnosis task and half chose the staging task in 2023. Regarding the algorithms, the studies mainly utilised deep CNNs (DCNN), with one article involving lightweight CNNs (LCNN) [35]. Eleven studies (11/27) used a two-stage design containing a tooth-identification or segmentation stage and a periodontitis-staging step [20, 30–32, 35, 36, 38, 42, 44, 47, 51]. Eight (8/27) studies utilised transfer learning [20, 21, 33, 39, 41, 45, 49, 51]. Reference tests were either experts’ direct opinions of periodontitis or their annotation of regions of interest (ROIs) based on different definitions. Sixteen studies (16/27) employed the new criteria proposed in the 2017 World Workshop on the Classification of Periodontal and Peri-Implant Diseases and Conditions [20, 29–34, 36–40, 42, 43, 45, 48], while one study (1/27) [41] used the International Workshop for Classification of Periodontal Diseases and Conditions (1999). Three studies (3/27) [28, 47, 52] carried out according to the World Health Organization’s standardized Community Periodontal Index (CPI) and four studies (4/27) [21, 44, 46, 49] roughly defined periodontitis based on the depth of bone resorption; the remaining two studies (2/27) [50, 51] did not mention the classification criteria. All studies compared the diagnostic performance of DL algorithms either with specialists or among different algorithms. More than two-thirds of articles (19/27) reported accuracy, while sensitivity, specificity, recall, precision, F1-score, ROC and AUROC were also reported among included studies.

**Table 2:** Characteristics of all included studies

First Author (publication year)	Country	Data sets	Modality	Machine learning algorithms	Study factor	Study factor definition	Application	Comparison if any	Main outcomes	Conclusions
**Q. Liu (2023)**	China	The 1924 images from the Second Affiliated Hospital were divided into training set (n = 1276), validation set (n = 376) and test set (n = 272). The 351 images from the Chinese Medicine Hospital were used as the second testing set.	Panoramic images	Alexnet	RBL	AAP/EFP 2018 classification; Stage I: AL of 1–2 mm; RBL < 15% (in the coronal third of the root); and no teeth loss due to periodontitis; Stage II: AL of 3–4 mm; 15%≤RBL ≤ 33% (in the coronal third of the root), and no teeth loss due to periodontitis; Stage III/IV: AL ≥ 5 mm; RBL > 33% (extending to the middle third of root and beyond). Healthy controls: ≤3 mm periodontitis disease; no AL; <10% BOP; no BL was assigned if the distance between CEJ and ABL was < 1.5 mm.	Automatically diagnose periodontitis with panoramic images.	Three blinded, experienced and calibrated periodontists	Accuracy: 0.800 Sensitivity: 0.820 Specificity: 0.780	DL methods can assist general dental practitioners in quickly and accurately diagnosing periodontitis.
**Chin-Chang Chen (2023)**	China (Taiwan)	8000 images from 270 subjects	Periapical images	Mask R-CNN	RBL	AAP/EFP 2017 classification.	Detect RBL.	Dentists	AP: 77.98	The proposed DL-trained ensemble model provides a critical cornerstone for radiographic detection and a valuable adjunct to periodontal diagnosis.
**Amasya (2023)**	Turkey	6000 images for training, about 100 images for testing.	Panoramic images	Cascade R-CNN	BL	AAP/EFP 2017 classification; Stage 1 indicates < 15% bone loss, Stage 2 indicates 15–33% bone loss, and further bone loss indicates, Stage 3 and 4. The threshold between Stages 3 and 4 is determined as 80% bone loss.	Diagnosis of periodontal defects on digital panoramic radiographs using a web-based AI software (DiagnoCat).	Three clinicians	Accuracy: 0.980, Precision: 0.971, Recall: 0.999, F-Score: 0.985	The use of a web-based AI software (DiagnoCat) can be beneficial in detecting PBL on panoramic radiographs.
**Jihye Ryu (2023)**	Korea	4083 images; five-fold cross-validation.	Panoramic images	Faster R-CNN with RPN	PBL	WHO CPI; Normal: confined level of BL up to CEJ; Moderate: PBL extending beyond CEJ but limited up to furcation of the tooth; Severe: PBL extending beyond the furcation of the tooth.	Detect PCT on panoramic radiographs.	Two trained dentists	Healthy: precision: 0.88, recall: 0.89, F1-score: 0.89. Periodontitis: precision: 0.86, recall: 0.84, F1-score: 0.85.	The regional grouping of teeth exhibited reliable detection performance for PBL using a large dataset, indicating the possibility of automating the diagnosis of periodontitis using panoramic images.
**I-Hui Chen (2023)**	China (Taiwan)	336 images (teeth: 390), training dataset (n = 82, teeth: 123), a validation dataset (n = 20) and test dataset (n = 336, teeth:390).	Periapical images	U-Net and Mask-RCNN	PBL	AAP/EFP 2017 classification; stage I: ABLD was < 15% (in the coronal third of the root); stage II: the ABLD was between 15% and 33.3% (in the coronal third of the root); stage III: the ABLD was > 33.3% (extending to the middle third of the root and beyond).	Stage the periodontitis by Length-based alveolar bone loss degree	Three independent calibrated board-certified periodontists	Accuracy: 72.8%	The proposed method can help dentists diagnose and monitor periodontitis progress on periapical radiographs.
**Zhengmin Kong (2023)**	China	1747 images, training set: validation set: test set = 7:1:2.	Panoramic images	PDCNN	RBL	AAP/EFP 2017 classification.	Automated RBL analysis to assist periodontitis diagnosis.	Professional dentists and the state-of-art architectures	Accuracy: 0.762 ± 0.003.	The proposed method successfully improves the RBL detection performance.
**Kubilay Muhammed Sunnetci (2022)**	Turkey	1432 images, training set: test set = 8:2.	Panoramic images	AlexNet and SqueezeNet + SVM, EfficientNetB5	PBL	Not mention.	Determine whether the subject has a PBL or non-PBL.	Expert and AlexNet, SqueezeNet and EfficientNetB5	Accuracy: 0.814.	AlexNet + Linear SVM and SqueezeNet + Medium Gaussian SVM architectures are more successful than all other classifiers.
**Nektarios Tsoromokos (2022)**	The Netherlands	446 images training set (n = 327), validation set (n = 49), test set (n = 70).	Periapical images	CNN	ABL	ABL < 33%; ABL ≥ 33%.	Detecting ABL.	A dentist	Sensitivity: 0.96, specificity: 0.41, accuracy: 0.80.	A CNN-trained algorithm on radiographic images showed a diagnostic performance with moderate to good reliability to detect and quantify %ABL in periapical radiographs.
**Jennifer Chang (2022)**	USA, China (Taiwan)	6,219 proximal surfaces from 1,832 images of 236 patients. Fivefold cross-validation.	Periapical images	Inception V3	RBL	AAP/EFP 2017 classification; healthy: no RBL; stage I: RBL < 15%; stage II: RBL 15–33%; stage III/IV: RBL > 33%.	Determine the severity of RBL.	Three board-certified and calibrated periodontists	Mean sensitivity: 0.86 ± 0.03; mean specificity: 0.88 ± 0.03; mean positive predictive value: 0.88 ± 0.03; mean negative predictive value: 0.86 ± 0.02.	The application of deep machine learning for the detection of ABL yielded promising results in this study.
**Rini Widyaningrum (2022)**	Indonesia	1100 images (100 original images and 1000 augmented images), with 75% for training and validation and 25% for testing.	Panoramic images	Multi-Label U-Net and Mask R-CNN	RBL	Normal: No radiographic bone loss; Stage 1: RBL < 15%; Stage 2: RBL 15–33%; Stage 3: RBL extending to the mid-third of root and beyond, with loss of ≤ 4 teeth; Stage 4: RBL extending to the mid-third of root and beyond, with loss of ≥ 5 teeth.	Image segmentation for periodontitis detection and classification.	A dentist and a periodontist	Accuracy: 95%; recall (sensitivity): 0.88; F1-score: 0.87.	Multi-Label U-Net produced superior image segmentation to that of Mask R-CNN; Mask R-CNN exhibited superior performance for periodontitis diagnosis in comparison with the ground truth image.
**Ho Sun Shon (2022)**	Korea	CBNUH dataset was 1044 images with 87 original images; AIHub dataset was 4010 images; both datasets were divided into a training set (70%) and testing set (30%).	Panoramic images	U-Net and YOLOv5	PBL and CEJ boundaries	Stage 1: RBL of < 15%; Stage 2: RBL of 15-33%; Stage 3: RBL of ≥ 33%; Stage 4: corresponds to cases where the sum of tooth loss and implant is ≥ 4 in identical conditions as Stage 3.	U-Net: tooth segmentation; YOLOv5: tooth identification; The integration of the two models: periodontitis classification.	Dental specialists	Accuracy: 0.928; mean recall: 0.805(0.799–0.811); precision: 0.732 (0.716–0.745); F1-score: 0.696 (0.681–0.709).	The novel framework was thus shown to exhibit a relatively high level of performance, and the findings in this study are expected to assist dental specialists with detecting the periodontitis stage and subsequent effective treatment.
**Linhong Jiang (2022)**	China	640 panoramic radiographs, training set: test set = 8:2.	Panoramic images	U-Net and YOLO-v4 Head	Radiographic bone resorption	Stage 1: PBL < 15%; Stage 2: 15%≤PBL ≤ 33%; Stage 3: PBL > 33%.	U-Net: tooth segmentation; CSPDarkNet, SPP + PAN, and YOLO-v4 Head: tooth identification; The integration of the two parts: periodontitis classification.	Three periodontists, each with more than 3 years of clinical experience	Accuracy: 0.77; precision: 0.77; sensitivity: 0.77; specificity:0.88; F1: 0.77.	It is feasible to establish DL model for assessment and staging radiographic periodontal ABL using two-stage architecture based on UNet and YOLO-v4.
**Tanjida Kabir (2022)**	USA	116 panoramic images, 682 periapical and bitewing radiographs, training set: validation set = 8:2, testing set: 55 additional periapical radiographs.	Periapical images	U-Net and U-Net with ResNet-34	RBL	Stage 1: RBL < 15% (in the coronal third of the root); Stage 2: 15%≤RBL ≤ 33% (in the coronal third of the root); Stage 3: RBL > 33% (extending to the middle third of root and beyond).	ABL assessment and periodontal diagnosis based on intraoral radiographs.	Three experts (two board-certified periodontists and one resident in the periodontics program)	Stage I RBL: sensitivity and specificity were 0.99, 0.93, respectively; Stage II RBL: sensitivity and specificity were 0.95, 0.66, respectively; Stage III RBL: sensitivity and specificity were 0.92, 0.88, respectively.	The proposed framework can correctly specify detailed diagnostic information associated with a single tooth without human intervention.
**Kübra Ertaş (2022)**	Turkey	144 patients, ten-fold cross-validation.	Panoramic images	DenseNet121, EfficientNetB0, InceptionV3, ResNet50, and VGG16	Periodontitis	Stage I: PD ≤ 4 mm, CAL ≤ 1–2 mm, horizontal BL, and no tooth loss due to periodontitis. Stage II: PD ≤ 5 mm, CAL ≤ 3–4 mm, horizontal BL, and no tooth loss due to periodontitis; Stage III: PD ≥ 6 mm, CAL ≥ 5 mm, and may have vertical BL and/or furcation involvement of class II or III, loss of ≤ 4 teeth due to periodontitis; Stage IV: PD ≥ 6 mm, CAL ≥ 5 mm, and may have vertical BL and/or furcation; involvement of class II or III, < 20 teeth may be present, and there is the potential for loss of ≥ 5 teeth due to periodontitis.	Perform the staging and grading of periodontitis only using Photographs.	DenseNet121, EfficientNetB0, InceptionV3, ResNet50, and VGG16	ResNet50 + SVM: accuracy: 0.882; F1: 0.872; precision: 0.864; recall 0.882.	The machine learning-based decision system presented herein can facilitate periodontal diagnoses despite its current limitations.
**Ghala Alotaibi (2022)**	Saudi Arabia	1724 intraoral periapical images, training dataset (n = 1206; 70%), validation dataset (n = 345; 20%), test dataset (n = 173; 10%).	Periapical images	VGG16	RBL	AAP 1999.	Detecting ABL in incisor teeth in periapical radiographs and the severity of the BL in the PCT.	Three independent and calibrated examiners, including a periodontist	Accuracy (binary classification): 73.04% Accuracy (multi-classification): 59.42%	This study revealed that the deep CNN algorithm (VGG-16) was useful to detect ABL in periapical radiographs, and has a satisfactory ability to detect the severity of bone loss in teeth.
**Haoyang Li (2021)**	China	Suzhou dataset: 298 panoramic radiographs; Zhongshan dataset: 204 panaramic radiographs. Randomly extracted 80% and 80% of Suzhou and Zhongshan data sets, respectively, as two training sets and the rest 20% and 20% were two testing sets, respectively.	Panoramic images	Mask R-CNN	ABL	No periodontitis: none of teeth has BL. Mild periodontitis: at least the ABL of one tooth is less than 15%; Moderate periodontitis: at least the ABL of one tooth is less than 33% and larger than 15%; Severe periodontitis: at least the ABL of one tooth is larger than 33%.	Detecting, numbering, and segmenting teeth and classifying the severity of periodontitis.	Two dentists	Suzhou dataset: accuracy: 0.892; F1-score: 0.889; Zhongshan dataset: accuracy: 0.812; F1-score: 0.819.	The entire architecture could not only outperform state-of-the-art methods and show robustness on two data sets in both periodontitis prediction, and teeth numbering and segmentation tasks, but also be interpretable for doctors to understand the reason why Deetal-Perio works so well.
**Raymond P. Danks (2021)**	UK	340 periapical radiographs were divided into training, validation, and test set.	Periapical images	Hourglass networks	PBL	BSP 2017 classification stage 1: PBL less than 15%; stage 2: PBL between 15 and 33%; stage 3: PBL between 33 and 67%; stage 4: PBL greater than 67%.	Automatically determine the severity stage and the regressive percentage of PBL by predicting the localization of the dental landmarks.	Two postgraduate specialist trainees in periodontology	Accuracy: 58%.	The system showed a promising capability to localise landmarks and estimate PBL on periapical radiographs.
**Matvey Ezhov (2021)**	USA, Turkey	Trainning and validation sets: localization datasets: 99 CBCT scans with the precisely segmented alveolar bone area and 120 CBCT scans with precisely segmented enamel area of teeth; classifcation (descriptor) datasets: 1135 CBCT scans. Test set: 30 CBCT maxillofacial images.	CBCT images	U-Net with CNN	ABL	Three BL types of different severity by calculating distances between pairs of periodontium landmarks segmented by a separate landmark localizer.	Detects and evaluates ABL in close vicinity to a tooth to classify different types of periodontitis.	Experienced dentomaxillofacial examiners	Periodontal bone loss: sensitivity and specificity were 0.9489 and 0.9661 respectively; Mild periodontal bone loss: sensitivity and specificity were 0.9321 and 0.9742 respectively; Moderate periodontal bone loss: sensitivity and specificity were 0.9111 and 0.9866 respectively; Severe periodontal bone loss: sensitivity and specificity were 0.9286 and 0.996 respectively.	The proposed AI system (Diagnocat) signifcantly improved the sensitivity and specifcity in regards to diagnosing the dental pathologies in comparison to human observers using CBCT imaging.
**Chun-Teh Lee (2021)**	USA	693 periapical images, training set: validation set: test set = 7:1:2. 644 additional periapical images for model evaluation.	Periapical images	U-Net and ResNet-34	RBL	Stage I: RBL < 15%(in the coronal third of the root); Stage II: 15%≤ RBL ≤ 33% (in the coronal third of the root); Stage III: extending to the middle third of the root and beyond (RBL > 33%); No BL (stage 0) was assigned if the distance between the CEJ and alveolar bone level is less than 1.5 mm disregarding the RBL percentage.	Alveolar bone level assessment and periodontal diagnosis based on intraoral radiographs.	Two periodontists and one periodontal resident	Stage I RBL: sensitivity, specificity, and accuracy were 0.82, 0.97, 0.91, respectively; Stage II RBL: sensitivity, specificity, and accuracy were 0.93, 0.86, 0.88, respectively; Stage III RBL: sensitivity, specificity, and accuracy were 0.80, 0.99, 0.99, respectively; No bone loss: sensitivity, specificity, and accuracy were 0.96, 1.00, 0.99, respectively.	The proposed DL model provides reliable RBL measurements and image-based periodontal diagnosis using periapical radiographic images.
**Hu Chen (2021)**	China	2900 periapical radiographs, five-fold cross-validation.	Periapical images	Faster R-CNNs	Periodontitis with bone resorptions	Periodo-mild: the bone resorption depth less than 1/3 of the tooth root length; Periodo-moderate: the bone resorption depth between 1/3 and 1/2 of the tooth root length; Periodo-severe: the bone resorption depth larger than 1/2 of the tooth root length.	Draws minimum bounding boxes to frame periodontitis with bone resorptions.	An expert dentist with more than 5 years of clinical experience	Periodo-Mild: Precision (0.4928 ± 0.0213), Recall (0.5555 ± 0.0173); periodo-Moderate: Precision (0.4298 ± 0.0361), Recall (0.4731 ± 0.0438); periodo-Severe: Precision (0.4746 ± 0.0426), Recall (0.4899 ± 0.0530).	The faster R-CNNs were able to detect periodontitis in dental periapical radiographs.
**Maira Moran (2021)**	Brazil	Training and validation sets: 1278 images of regions with PBL and 1344 images of healthy regions. The training–validation ratio was 80:20. Test set: 52 images of each class (with and without PBL), resulting in 104 regions.	Periapical images	ResNet and Inception	PBL	Horizontal BL consists of a horizontal loss in the alveolar bone’s height. Vertical BL can be identified as a deformity in the alveolus extending apically along the root of the affected tooth from the alveolar crest. The interproximal crater consists of a lesion that radiographically can be observed as a two-walled, trough-like depression. This loss has a band-like or irregular appearance in the interdental region between adjacent teeth.	Predict PBL.	Experienced dentists and different models	The accuracy for ResNetNearest, ResNetBilinear, ResNetBicubic, ResNetLanczos, ResNetSRCNN, ResNetSRGAN, InceptionNearest, InceptionBilinear, InceptionBicubic, InceptionLanczos, InceptionSRCNN, InceptionSRGAN were 0.654, 0.731, 0.740, 0.712, 0.769, 0.740, 0.788, 0.952, 0.817, 0.731, 0.721, and 0.750, respectively.	Both deep-learning methods, especially SRGAN, generate high-resolution images with high visual quality in aspects that influence PBL assessment, promoting easier diagnosis.
**Hyuk-Joon Chang (2020)**	Korea	330, 115, and 73 images were used to detect the PBL, the CEJL, and the teeth, respectively. The images were randomly separated into a training set (90%), and a test set (10%) before data augmentation. Ten panoramic images for evaluation, which were not used for detection.	Panoramic images	A modified CNN	PBL, CEJ level, and the teeth.	AAP/EFP 2017 classification Stage 1: RBL < 15% (in the coronal third of the root); Stage 2: RBL 15-33% (in the coronal third of the root); Stage 3: RBL > 33% (extending to the middle third of the root and beyond).	Detect the radiographic bone level (or the CEJ level).	Three OMF radiologists (a resident, a fellow and a professor).	N/A	The novel hybrid framework that combined DL architecture and the conventional CAD approach demonstrated high accuracy and excellent reliability in the automatic diagnosis of PBL and staging of periodontitis.
**Bhornsawan Thanathornwong (2020)**	Thailand	100 panoramic radiographs, training set: validation set: test set = 7:1:2.	Panoramic images	Faster R-CNNs	Periodontal status	Healthy: CAL < 3 mm; Moderately periodontally compromised: BOP and CAL < 6 mm or BL < 4 mm; Severely periodontally compromised: CAL > 6 mm and BL > 4 mm. Moderately and severely periodontally compromised teeth were grouped together to form the periodontally compromised teeth group.	Detect PCT.	Three experts in periodontology	Sensitivity: 0.84, specificity: 0.88, F-measure: 0.81.	The faster R-CNN trained on a limited amount of labeled imaging data performed satisfactorily in detecting PCT. The application of a faster R-CNN to assist in the detection of PCT may reduce diagnostic effort by saving assessment time and allowing automated screening documentation.
**Sevda Kurt Bayrakdar(2020)**	Turkey	2276 panoramic images, of which 1137 were of bone loss cases and 1139 were of periodontally healthy cases, regardless of gender. This dataset is divided into training (n = 1856), validation (n = 210), and testing (n = 210) sets.	Panoramic images	InceptionV3	Periodontal diseases including ABL	Radiographs showing bone resorption with a horizontal/vertical shape or bone defects were included in the BL group. Radiographs with no loss of bone crests or with the alveolar bone completely covering the root surfaces of the teeth (normal anatomical structure) were included in the periodontally healthy group.	Determine ABL and periodontal disease/health status from dental panoramic radiography images.	An oral and maxillofacial radiologist and a periodontologist	Sensitivity: 0.9429; specificity: 0.8857; precision: 0.8919; accuracy:0.9143; F1 score: 0.9167.	The CNN system successfully determines PBL. Therefore, it can be used to facilitate diagnosis and treatment planning by oral physicians in the future.
**Joachim Krois (2019)**	Germany	2001 cropped image segments from 85 panoramic images, training set (n = 1456), validation set (n = 353)	Panoramic images	CNNs	PBL	Not mention.	Detect PBL.	Six dental practitioners	The mean (SD) classifcation accuracy of the CNN was 0.81 (0.02). Mean (SD) sensitivity and specifcity were 0.81 (0.04), 0.81 (0.05), respectively.	A moderately complex CNN trained on a limited amount of labeled radiographic images showed at least similar diagnostic performance as experienced dentists to detect PBL.
**Jaeyoung Kim (2019)**	South Korea	12,179 panoramic dental radiographs, training set (n = 11,189), validation set (n = 190), test set (n = 800)	Panoramic images	DeNTNet	PBL	Not mention.	Predict the existence of PBL for each tooth, and provide teeth numberings of predicted lesions.	Five dental clinicians	Baseline: F1 score: 0.66; sensitivity: 0.66; specificity: 0.94; PPV: 0.65; NPV: 0.94.	The proposed model was able to achieve a PBL detection performance superior to that of dental clinicians.
**Jae-Hong Lee (2018)**	Korea	1740 periapical radiographic dataset, training set (n = 1,044), validation set (n = 348), test set (n = 348)	Periapical images	VGG-19	PCT	Healthy: CAL < 3 mm; Moderate PCT: bleeding on probing and CAL < 6 mm or a BL < 4 mm; Severe PCT: CAL > 6 mm and a BL > 4 mm.	Evaluate the potential usefulness and accuracy of this system for the diagnosis and prediction of PCT.	Three calibrated board-certified periodontists	For premolars: accuracy: 82.8% (95% CI, 70.1–91.2%); For molars: accuracy: 73.4% (95% CI, 59.9–84.0%).	The deep CNN algorithm was useful for assessing the diagnosis and predictability of PCT.

DL, deep learning; ML, machine learning; CNN, convolutional neural network; RPN, region proposal network; PDCNN, CNN-based periodontitis detection network; DeNTNet, deep neural transfer network; SVM, support vector machines; YOLO, you only look once; AI, artificial intelligence; SRGAN: super-resolution generative adversarial network; CAD, computer aided diagnoses; WHO, The World Health Organization; CPI, Community Periodontal Index; AAP/EFP, The American Academy of Periodontology and European Federation of Periodontology; AAP 1999, The 1999 International Workshop for a Classification of Periodontal Diseases and Conditions; BL, bone loss; RBL, radiographic bone loss; PBL, periodontal bone loss; ABL, alveolar bone loss; ABLD, alveolar bone loss degree; PCT, periodontally compromised teeth; CEJ, cemento-enamel junction; BOP, bleeding on probing; AL, attachment level; CAL, clinical attachment level; PD, probing depth; CBCT, cone-beam computed tomography; PPV, positive predictive value; NPV, negative predictive value; SD, standard deviation; AP, average precision

### Meta-analysis

From the 27 articles selected for the systematic review, 14 were excluded from the subsequent meta-analysis because TP, FN, FP and TN were not reported and could not be calculated. Consequently, 13 studies were included in the meta-analysis [21, 29, 33–35, 40, 41, 43, 47, 49–52]. The correlation analysis showed heterogeneity due to the threshold effect (*r* = 0.13; *P* = 0.02). Therefore, instead of directly combining the sensitivity and specificity to demonstrate the overall accuracy, an SROC curve was generated (Supplementary Fig. 4). The AUSROC was 0.94 (95% confidence interval [95%CI] 0.91–0.96). To investigate the source of heterogeneity, we conducted an influence analysis (Supplementary Fig. 5). Supplementary Fig. 5(c) and Supplementary Fig. 5(d) both indicated that the seventh article was an outlier [43], which can affect the stability of the results. When this article was removed, the threshold effect disappeared (*r* = − 0.45; *P* = 0.20), and the combined sensitivity, specificity, positive LR, negative LR and DOR were 0.88 (*95%CI* 0.82–0.92), 0.82 (*95%CI* 0.72–0.89), 4.9 (*95%CI* 3.2–7.5), 0.15 (*95%CI* 0.10–0.22) and 33 (*95%CI* 19–59), respectively.

**Fig. 2:**
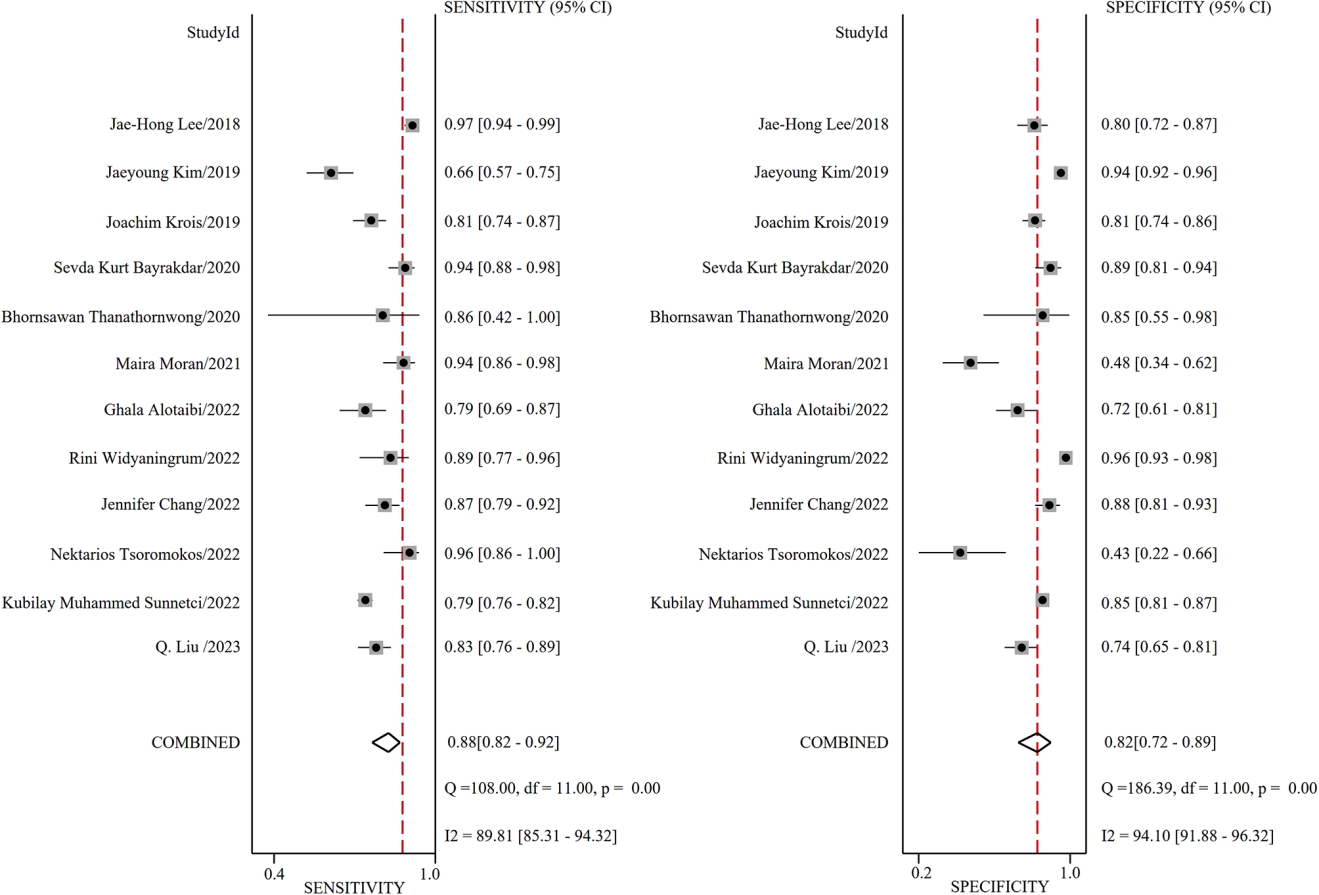
The forest plot for sensitivity and specificity of deep learning for periodontitis diagnosis

**Fig. 3:**
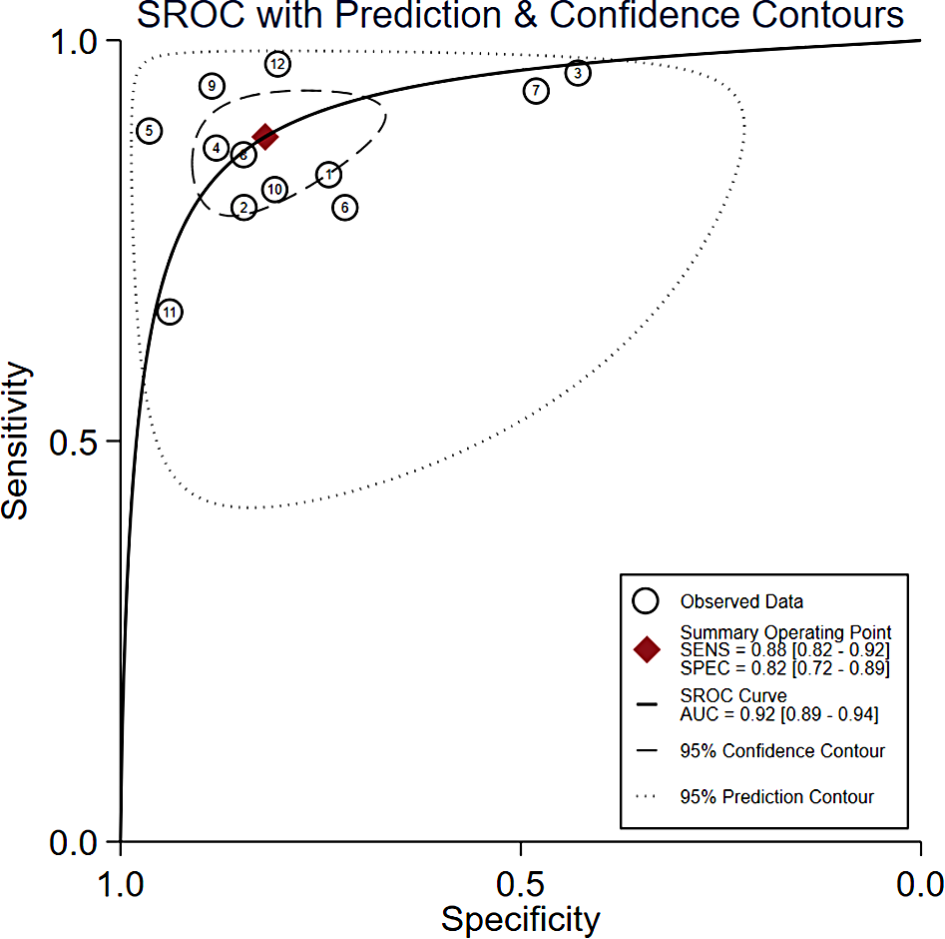
The summary receiver operating characteristic curve of diagnostic accuracy of periodontitis by deep learning excludes the seventh article. SENS, sensitivity; SPEC, specificity; SROC, summary receiver operating characteristic; AUC, area under curve

**Fig. 4:**
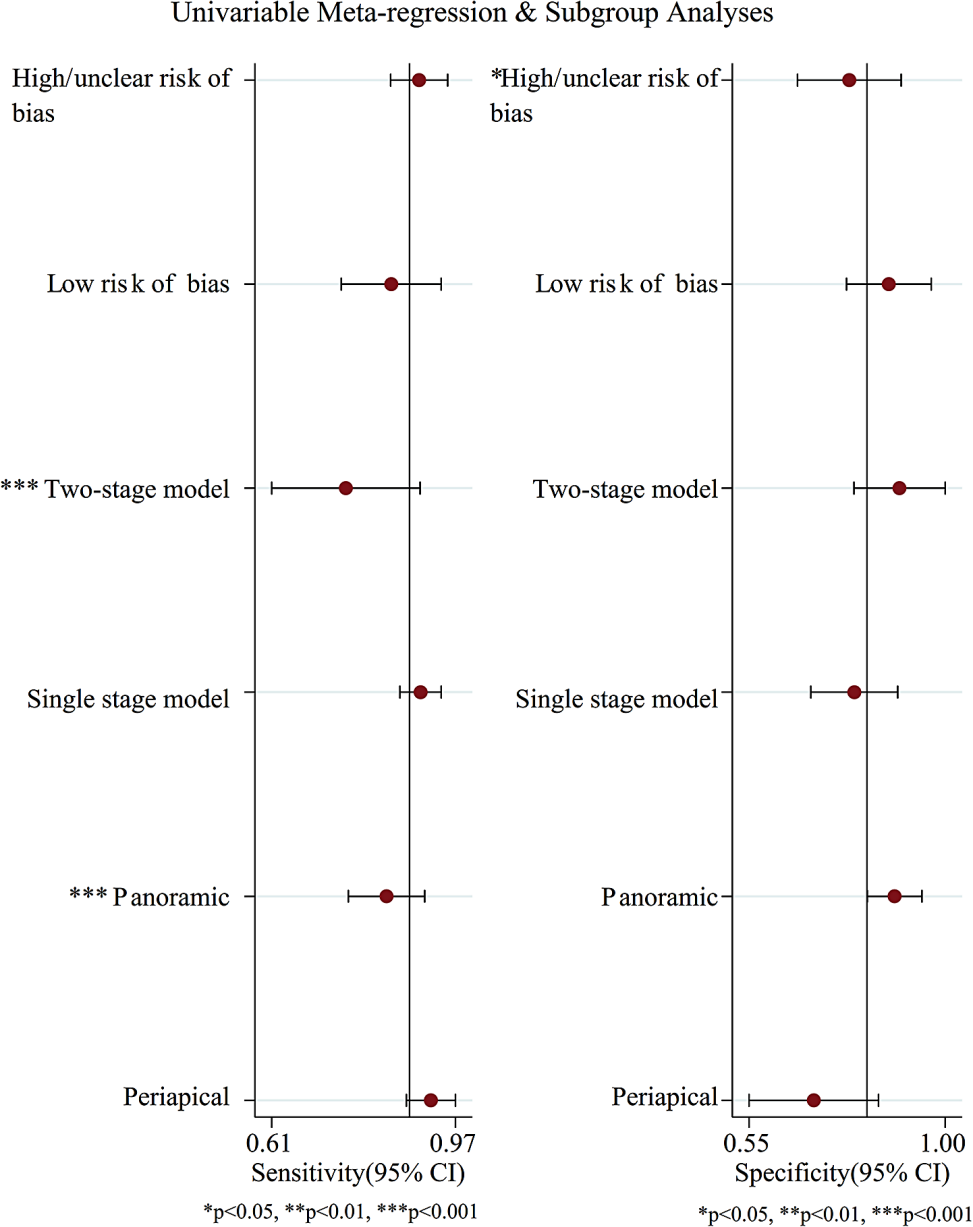
Subgroup analysis based on article quality, dental image modality and model type

**Fig. 5:**
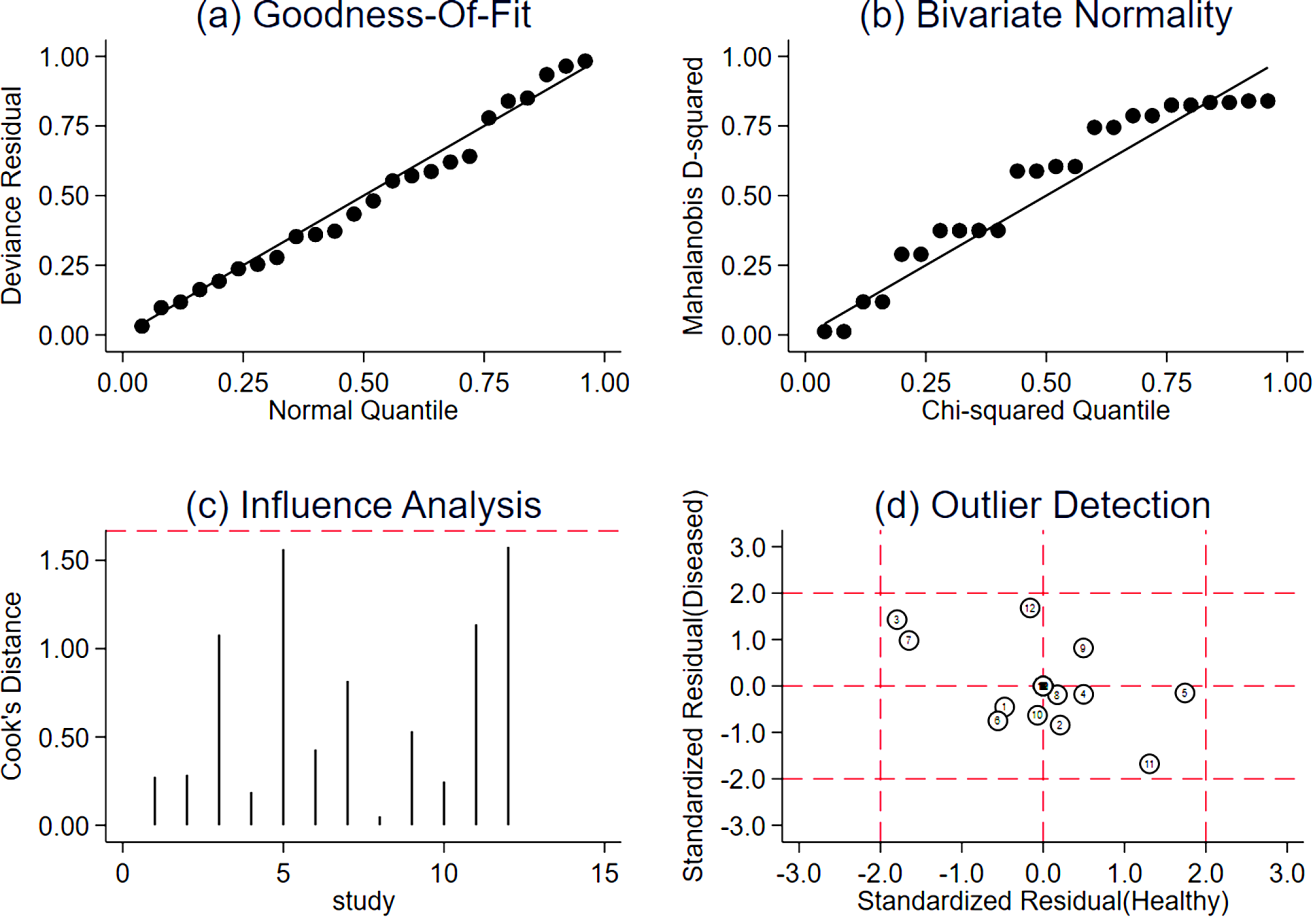
Influence analysis exclude the seventh article

**Fig. 6:**
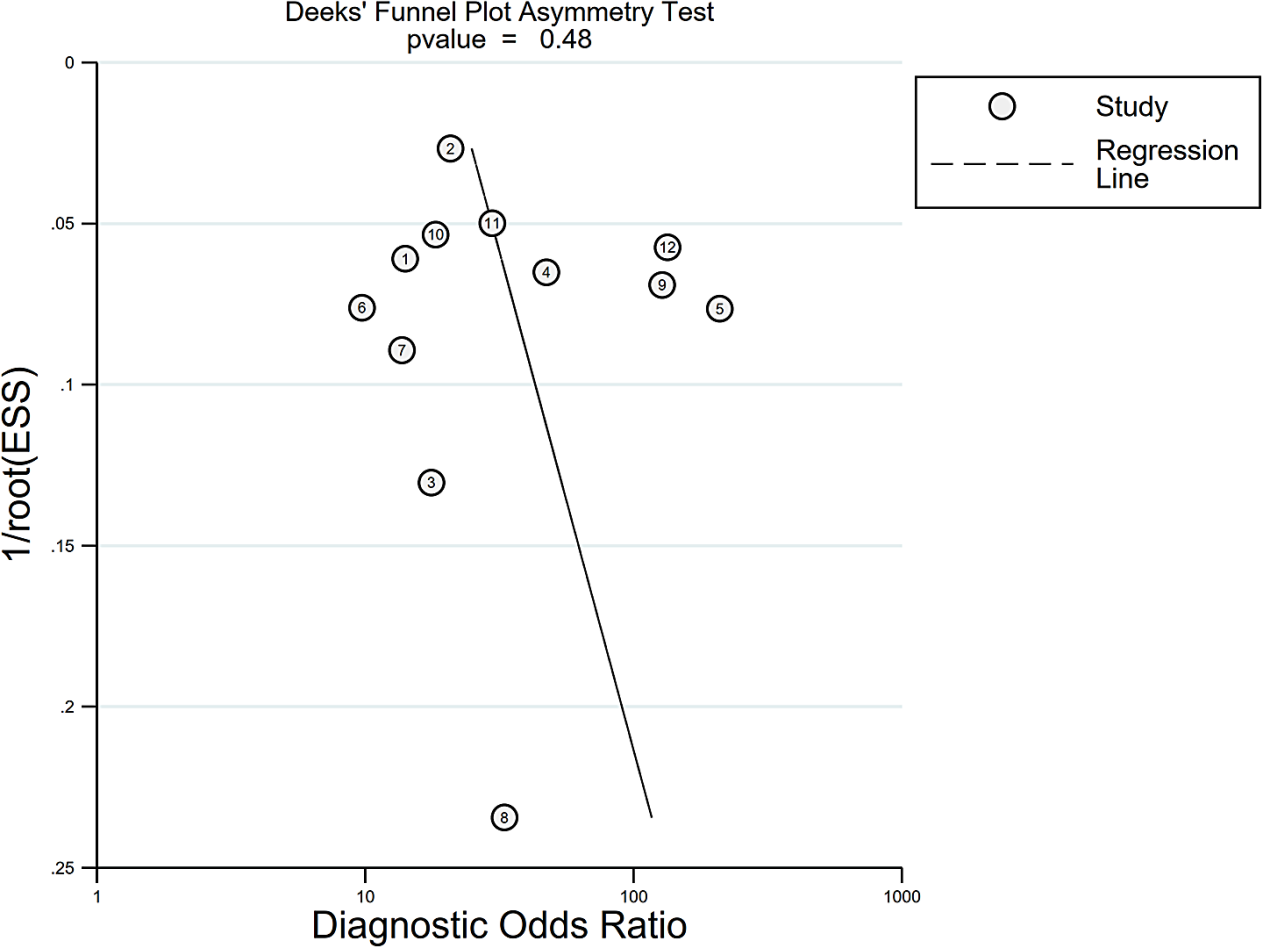
Publication bias of periodontitis diagnosis by deep learning

Figure 2 illustrates the forest plot of sensitivity and specificity of the DL algorithms for the periodontitis classification. The AUSROC (Fig. 3) was 0.92 (*95%CI* 0.89–0.94), which implied that the diagnostic test had high accuracy. According to the Fagan nomogram (Supplementary Fig. 6), the prior probability of this diagnostic test was 50%, the positive LR was 6, the posterior probability after a positive test was 85%, and the negative LR was 0.10. The posterior probability after a negative test was 9%. The subgroup analysis results showed that heterogeneity of sensitivity was statistically significant in model type and dental image modality, and heterogeneity of specificity was statistically significant in article quality (Fig. 4). In detail, a single model would get a significantly higher sensitivity than a two-stage model (*P* < 0.01). Moreover, the modality of dental images may cause heterogeneity of sensitivity (*P* < 0.01). Diagnosis sensitivity based on periapical images was higher than that on panoramic images. Furthermore, articles scored as high or unclear risk of bias would get a significantly lower specificity than low risk of bias articles (*P* = 0.03). Both meta-regression results indicate that there is no statistically significant correlation between sample size and sensitivity (*P* = 0.069), as well as between sample size and specificity (*P* = 0.252) (Supplementary Fig. 7, Supplementary Fig. 8). The influence analysis demonstrated that the results were stable by removing one study at a time (Fig. 5). Deeks’ funnel plot asymmetry test illustrated no publication bias (*t* = 0.74, *P* = 0.48) (Fig. 6).

## Discussion

In this systematic review, we compiled and evaluated studies that utilised DL methods to classify periodontitis based on dental images. With the rise of DL technology, an increasing number of articles have been published on the intersection of periodontitis classification and DL, especially in 2022. The overall quality of the included studies was limited, more high-quality studies are urgently needed. In addition, more than half of the included articles reported that the accuracy, sensitivity, and specificity of their algorithms for classifying periodontitis were > 0.8. The SROC curve also showed the high accuracy of the DL methods for classification. The study by Lee et al. [43], which reported the specificity as 1 for distinguishing non-periodontitis individuals, was an outlier in our meta-analysis. Moreover, the Fagan nomogram indicated that when a DL method classifies a positive result, there is a high probability of periodontitis, and if the classification is negative, the probability of periodontitis is low. These findings are further discussed in the following sections.

### Characteristics of dental images

There are very few large and high-quality public databases of dental radiographs. Consequently, dental radiographs must be manually labeled, which is time-consuming and needs to be urgently addressed. Random shift augmentation, oversampling, adjusting weights in the loss function, and transfer learning were used to overcome class-imbalanced issues, which detrimentally contributed to DL classification performance [30, 39, 41, 42, 50, 51, 53].

In terms of modalities of dental images, the studies included in our analysis predominantly used periapical images, panoramic images and CBCT images for periodontitis classification. Nine studies detected RBL in periapical radiograph images. Periapical radiograph images capture the teeth and the surrounding alveolar bone, and therefore can fully provide information on RBL. However, the view of this modality is small, with only three to four teeth on a single image [54]. Over half of the studies in our analysis detected RBL in panoramic X-ray images, which show the whole mouth. However, as two-dimensional modalities, both periapical radiograph images and panoramic X-ray images cannot provide three-dimensional information and have problems with geometric distortion and anatomic noise [55]. All these limitations may affect the performance of periodontitis classification. Only one study in our analysis used CBCT and did detect RBL in the resulting images [44]. Although CBCT can provide three-dimensional information, there are still some limitations caused by artifacts, noise and poor soft tissue contrast [56]. Consequently, dental image processing plays a vital role in periodontitis classification.

### Processing of dental images

Two aspects should be considered for an accurate periodontitis classification. One is the quality of dental images, and the other is model performance. To deal with image quality problems, the included articles employed super-resolution and noise reduction methods. One study conducted in Brazil reconstructed high-resolution images from low-resolution images by using four conventional interpolation methods (nearest, bilinear, bicubic, Lanczos) and two DL methods (super-resolution CNN and a variation of the super-resolution generative adversarial network) [21]. Two studies used the contrast-limited adaptive histogram equalization technique for image denoising [39, 40]. Besides noise reduction, one study conducted in the USA also introduced a series of processes to precisely draw the contour of bone, tooth, and cemento-enamel junction after model prediction to improve model performance [43]. In addition, a quarter of the studies resized and normalised the images to improve model performance. Furthermore, because obtaining dental images is difficult, almost half of the included articles used data augmentation techniques to increase the number of images [48, 50, 52].

### Classification using dental images

Regarding the task of classification using DL models, classical models such as U-Net and YOLO were often utilised in the included studies [57, 58], regardless of the specific diagnosis task chosen. For tasks involving a two-stage design, U-Net was typically used for segmenting ROIs, while YOLO was employed for object detection. U-Net has been proven to quickly and accurately identify targets in medical images and generate high-quality segmentation results [59]. Additionally, the structure of U-Net can be flexibly adjusted according to the specific needs of the task [59]. Various versions of YOLO, from YOLOv3 to YOLOv5, have been utilised based on different study purposes. Feature Pyramid Network (FPN) was also employed for the ROI segmentation stage [60]. FPN fuses multi-layered features and makes predictions at each fused feature layer, thus, it shows significant improvement in small-object detection without considerably increasing computation. Faster region-based CNN (Faster R-CNN) combines a Region Proposal Network (RPN) and a Fast R-CNN that shares full-image convolutional features to overcome the computational problem, which is why Faster R-CNN is popular in periodontitis diagnosis [61]. Mask R-CNN, which is an extension of Faster R-CNN, has also been employed [62]. Danks et al. employed a symmetric hourglass network that can capture every scale information and combine them to make the final predictions [45].

Based on the included publications, transfer learning is an efficient method for training datasets with limited samples, and it can enhance the model training efficiency. In addition, using appropriate regularisation methods can improve model performance.

### Strengths and limitations

#### Strengths

1. The strength of this review is that we systematically summarised and evaluated the studies on DL for periodontitis classification based on dental images. Moreover, we have described the development trend of DL technology in the field of periodontitis.
2. In addition, we used meta-analysis to quantitatively evaluate the threshold effect and heterogeneity of the included articles and analysed the possible sources of heterogeneity in detail.

#### Limitations

1. DL-based periodontitis classification is an emerging field and most studies conducted thus far have predominantly focused on Asian populations. This limited regional focus has resulted in a constrained sample representation, thereby impacting the external validity of the findings.
2. Except for three articles that utilised publicly available databases, the samples in the other studies were solely derived from hospital settings, thereby lacking representation from community-based data.
3. No study described the demographic information pertaining to the included subjects. Considering that demographic information could potentially influence the severity of periodontitis and consequently contribute to the heterogeneity observed, it is essential to address this aspect in future research.
4. Only three studies incorporated an external dataset to assess the performance of DL-based models. In contrast, all the other studies relied on training and testing datasets derived from the same source, potentially limiting the generalisability of their results.
5. Since the gold standard of periodontitis diagnosis and classification should be clinical attachment loss (CAL), it would lead to underestimation of periodontal status only based on RBL. However, the classification is still important in the clinical practice when the direct evidence (CAL) is not available.

## Conclusions

In summary, the accuracy of DL is high for classifying periodontitis based on dental images. DL is an efficient approach to reducing the workload of dentists and the time consumed during clinical practice. Furthermore, the various DL models have their advantages and disadvantages, and the choice of model should be based on the specific task objectives and requirements. Future research should be designed rigorously to reflect the DL truth performance. The optimisation of DL architecture can promote the performance of periodontitis classification with dental images. Moreover, improving dental image quality and performing regularisation can yield higher periodontitis diagnostic accuracy. In addition, data imbalance is an issue that needs to be considered to enhance diagnostic performance.

### Electronic supplementary material

Below is the link to the electronic supplementary material.

Supplementary Table 1: Database search strategy

Supplementary Table 2: Quality assessment of included studies (n = 27)

Supplementary Table 3: Summary of quality of evidence based on Grading of Recommendations Assessment, Development and Evaluation (GRADE)

Supplementary Figure 1

Supplementary Figure 2

Supplementary Figure 3

Supplementary Figure 4

Supplementary Figure 5

Supplementary Figure 6

Supplementary Figure 7

Supplementary Figure 8

## Data Availability

The datasets used and/or analyzed during the current study are available from the corresponding author on reasonable request.

## Abbreviations

DALYs: Disability-adjusted life years
GBD: Global Burden of Diseases
ML: Machine learning
DL: Deep learning
CNNs: Convolutional neural networks
CBCT: Cone-beam computed tomography
PIRD: P = population, I = index test, R = reference test, D = diagnosis of interest
PRISMA-DTA: Preferred Reporting Items for Systematic Reviews and Meta-analyses for Diagnostic Test Accuracy Studies
PPV: Positive predictive values
NPV: Negative predictive values
ROC: Receiver operating characteristic curve
AUC: The area under the curve
AUROC: The area under the receiving operating characteristic curve
IoU: Intersection over union
PA: Pixel accuracy
AP: Average precision
ARR: Average recall rate
AI: Artificial intelligence
QUADAS-2: Quality Assessment of Diagnostic Accuracy Studies
TP: True positive
FP: False positive
TN: True negative
FN: False negative
LR: Likelihood ratio
DOR: Diagnostic odds ratio
SROC: Summary receiver operating characteristic
AUSROC: Area under summary receiver operating characteristic
DCNN: Deep convolutional neural networks
LCNN: Lightweight convolutional neural networks
RBL: Radiographic bone loss
ROIs: Regions of interest
FPN: Feature Pyramid Network
Faster R-CNN: Faster region-based CNN
RPN: Region Proposal Network
