# Supplementary material for: Deep learning for classifying the stages of periodontitis on dental images: a systematic review and meta-analysis
Source: BMC Oral Health. 2023 Dec 19;23:1017. doi: 10.1186/s12903-023-03751-z (PMC10729340; doi:10.1186/s12903-023-03751-z)
Supplement: Supplementary file 1 — Supplementary Table 1: Database search strategy [file 12903_2023_3751_MOESM1_ESM.docx]

| **Supplementary Table 1. Database search strategy** | |
| --- | --- |
| Database | Search strategy |
| EMBASE | ((‘periodontitis’/exp or ‘periodontitis’ or ‘periodontal disease’/exp or ‘periodontal disease’ or ‘periodontal status’) AND (‘image’/exp or ‘image’ or ‘image processing’/exp or ‘image processing’ or ‘computer assisted diagnosis’/exp or ‘computer assisted diagnosis’) AND (‘artificial intelligence’/exp or ‘artificial intelligence’ or ‘machine learning’/exp or ‘machine learning’ or ‘deep learning’/exp or ‘deep learning’ or ‘convolution neural network’/exp or ‘convolutional neural network’)) |
| Web of Science | ((((TS=(periodontitis)) OR TS=(periodontal disease)) OR TS=(periodontal status)) AND (((((TS=(image processing)) OR TS=(computer-aided diagnosis)) OR TS=(computer-based diagnosis)) OR TS=(smart diagnose)) AND (((((((TS=(artificial intelligence)) OR TS=(machine learning)) OR TS=(deep learning)) OR TS=(convolutional neural networks)) OR TS=(CNN)) OR TS=(CNNs))) |
| Scopus | (“periodontitis” or “periodontal disease” or “periodontal status”) AND (“image” or “image processing” or “computer-aided diagnosis” or “computer-based diagnosis” or “smart diagnose”) AND (“artificial intelligence” or “machine learning” or “deep learning” or “convolutional neural networks” or “CNN” or “CNNs”)) |
| PubMed | ((periodontitis or periodontal disease or periodontal status) AND (image or image processing or computer-aided diagnosis or computer-based diagnosis or smart diagnose)) AND (artificial intelligence or machine learning or deep learning or convolutional neural networks or CNN or CNNs) |
| Google Scholar | ((periodontitis or periodontal disease or periodontal status) AND (image or image processing or computer aided diagnosis or computer-based diagnosis or smart diagnose) AND (artificial intelligence or machine learning or deep learning or convolutional neural networks or CNN or CNNs)) |
