# Supplementary material for: Deep learning for classifying the stages of periodontitis on dental images: a systematic review and meta-analysis
Source: BMC Oral Health. 2023 Dec 19;23:1017. doi: 10.1186/s12903-023-03751-z (PMC10729340; doi:10.1186/s12903-023-03751-z)
Supplement: Supplementary file 2 — Supplementary Table 2: Quality assessment of included studies (n = 27) [file 12903_2023_3751_MOESM2_ESM.docx]

**Supplementary Table S2.** Quality assessment of included studies (n = 27)

| **Study** | **Risk of Bias** | | | | **Applicability Concerns** | | |
| --- | --- | --- | --- | --- | --- | --- | --- |
|  | patient selection | Index Test | Reference Standard | Flow and Timing | patient selection | Index Test | Reference Standard |
| Chin-Chang Chen (2023) | unclear | low | low | low | low | low | low |
| Amasya (2023) | unclear | low | low | low | low | low | low |
| Q. Liu (2023) | high | low | low | low | low | low | low |
| Jihye Ryu (2023) | low | low | high | low | low | high | high |
| I-Hui Chen (2023) | high | low | low | low | low | high | high |
| Zhengmin Kong(2023) | unclear | low | low | low | low | low | low |
| Kubilay Muhammed Sunnetci(2022) | unclear | unclear | low | low | low | high | high |
| Nektarios Tsoromokos(2022) | unclear | low | low | low | low | low | low |
| Jennifer Chang(2022) | low | low | low | low | low | low | low |
| Rini Widyaningrum(2022) | low | low | low | low | low | low | low |
| Ho Sun Shon(2022) | unclear | low | low | low | low | low | low |
| Linhong Jiang(2022) | unclear | low | low | low | low | low | low |
| Tanjida Kabir(2022) | unclear | low | low | low | low | low | low |
| Kübra Ertaş(2022) | low | unclear | low | low | low | low | low |
| Ghala Alotaibi(2022) | low | low | low | low | low | low | low |
| Haoyang Li(2021) | unclear | low | high | low | low | low | low |
| Raymond P. Danks(2021) | unclear | low | low | low | low | low | low |
| Matvey Ezhov(2021) | low | low | low | low | low | low | low |
| Chun-Teh Lee (2021) | low | low | low | low | low | low | low |
| Hu Chen（2021） | low | low | low | low | low | low | low |
| Maira Moran（2021） | low | unclear | high | low | low | high | high |
| Hyuk-Joon Chang(2020) | unclear | low | low | low | low | low | low |
| Bhornsawan Thanathornwong(2020) | low | low | low | low | low | low | low |
| Sevda Kurt Bayrakdar(2020) | low | unclear | high | low | low | high | high |
| Joachim Krois(2019) | low | low | low | low | low | high | high |
| Jaeyoung Kim(2019) | low | unclear | low | low | low | high | high |
| Jae-Hong Lee(2018) | unclear | unclear | low | low | low | low | low |
