# Supplementary material for: Deep learning for classifying the stages of periodontitis on dental images: a systematic review and meta-analysis
Source: BMC Oral Health. 2023 Dec 19;23:1017. doi: 10.1186/s12903-023-03751-z (PMC10729340; doi:10.1186/s12903-023-03751-z)
Supplement: Supplementary file 3 — Supplementary Table 3: Summary of quality of evidence based on Grading of Recommendations Assessment, Development and Evaluation (GRADE) [file 12903_2023_3751_MOESM3_ESM.docx]

**Supplementary Table S3** Summary of quality of evidence based on Grading of Recommendations Assessment, Development and Evaluation (GRADE)

| Model type | Dental image modality | No of studies | Design | Limitations (risk of bias) | Indirectness | Inconsistency | Imprecise data | Publication bias | outcomes | | Quality |
| --- | --- | --- | --- | --- | --- | --- | --- | --- | --- | --- | --- |
|  |  |  |  |  |  |  |  |  | sensitivity | specificity |  |
| single model | panoramic | 4 studies (1110 patients) | cross-sectional studies | high risk of bias ^a^ | not serious | not serious | serious ^c^ | Not detected | 0.88 [0.81, 0.92] | 0.8 [0.76, 0.94] | low |
|  | periapical | 5 studies (958 patients) | cross-sectional studies | high risk of bias ^a^ | not serious | serious ^b^ | serious ^c^ | Not detected | 0.92 [0.85,0.96] | 0.70[0.52,0.83] | very low |
| two-stage model | panoramic | 3 studies (2252 patients) | cross-sectional studies | high risk of bias ^a^ | not serious | serious ^b^ | serious ^c^ | Not detected | 0.75 [0.61,0.90] | 0.89[0.79,1.00] | very low |

^a^ Quality of evidence deducted from high to moderate due to high risk of bias or unclear risk of bias based on QUADAS-2

^b^ Statistically significant heterogeneity of results for sensitivity and specificity, reducing quality of evidence from moderate to low.

^c^ Wide confidence interval downgraded quality of evidence in single model with periapical images and two-stage model with panoramic images settings from low to very low, and in single model with panoramic images from moderate to low.
