# Supplementary figures and images for: Deep learning for classifying the stages of periodontitis on dental images: a systematic review and meta-analysis
Source: BMC Oral Health. 2023 Dec 19;23:1017. doi: 10.1186/s12903-023-03751-z (PMC10729340; doi:10.1186/s12903-023-03751-z)

## Risk of bias

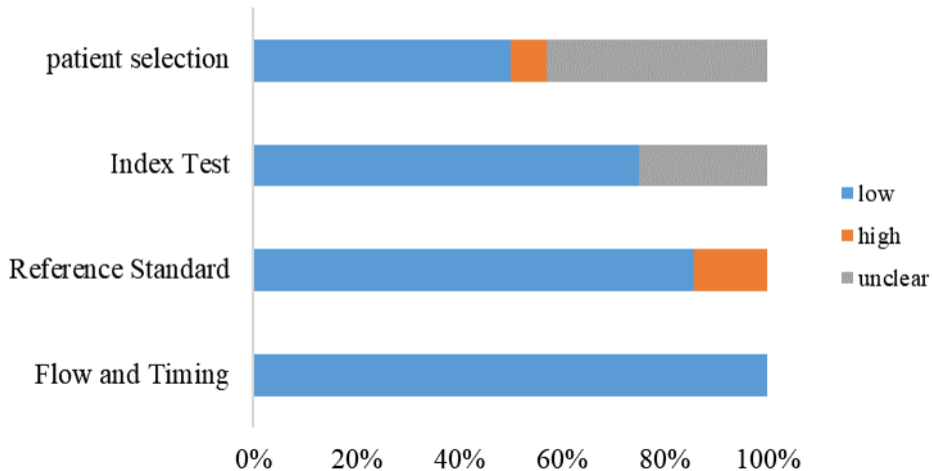

Supplement: Supplementary file 4 — Supplementary Figure 1 [file 12903_2023_3751_MOESM4_ESM.pdf]

## Applicability Concerns

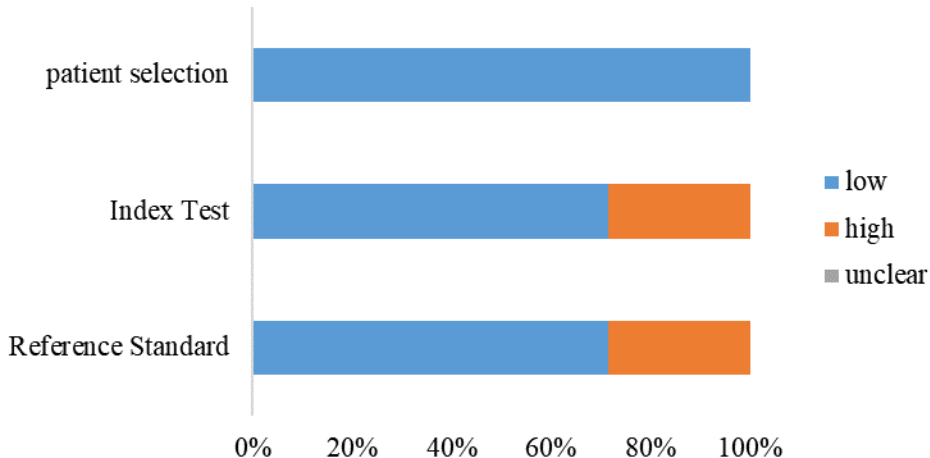

Supplement: Supplementary file 5 — Supplementary Figure 2 [file 12903_2023_3751_MOESM5_ESM.pdf]

Temporal Trend of Article Counts

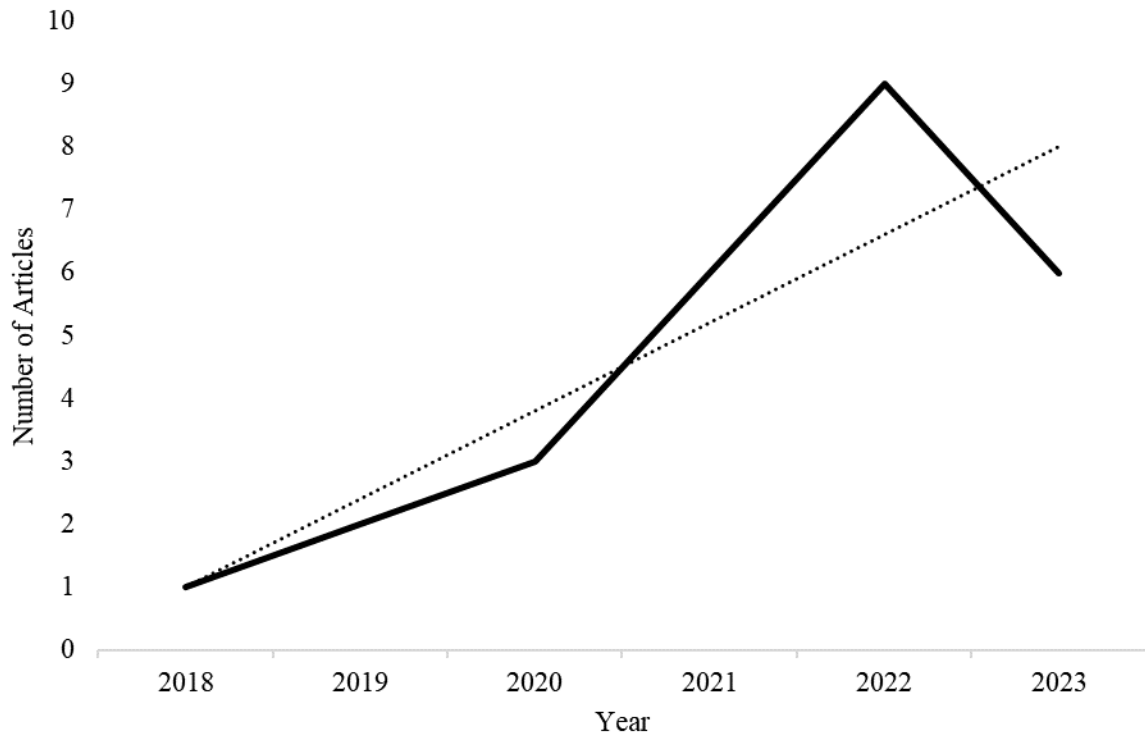

Supplement: Supplementary file 6 — Supplementary Figure 3 [file 12903_2023_3751_MOESM6_ESM.pdf]

# SROC with Prediction & Confidence Contours

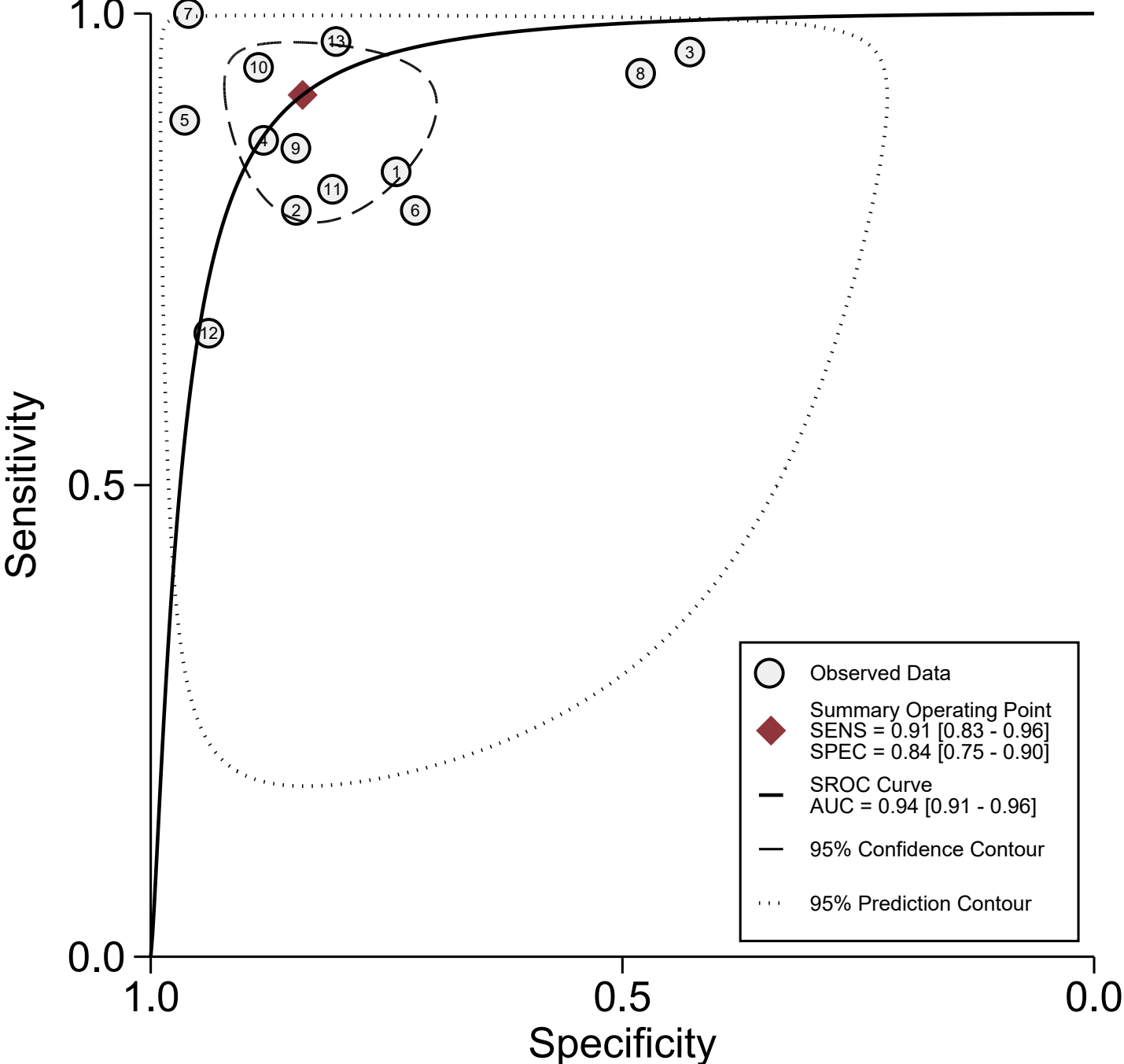

Supplement: Supplementary file 7 — Supplementary Figure 4 [file 12903_2023_3751_MOESM7_ESM.pdf]

(a) Goodness-Of-Fit

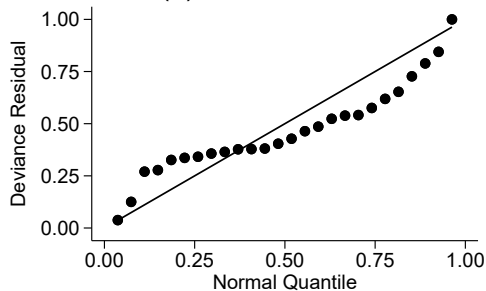

(b) Bivariate Normality

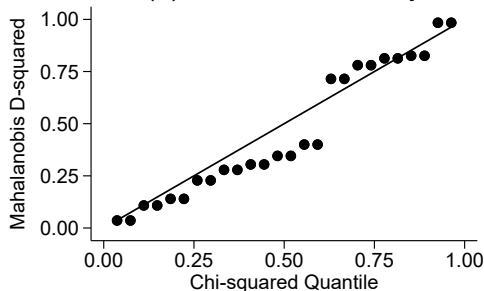

(c) Influence Analysis

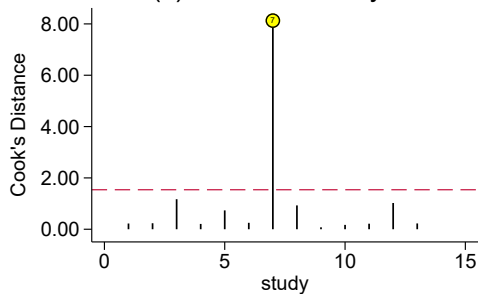

(d) Outlier Detection

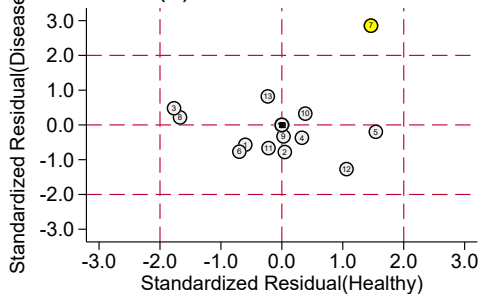

Supplement: Supplementary file 8 — Supplementary Figure 5 [file 12903_2023_3751_MOESM8_ESM.pdf]

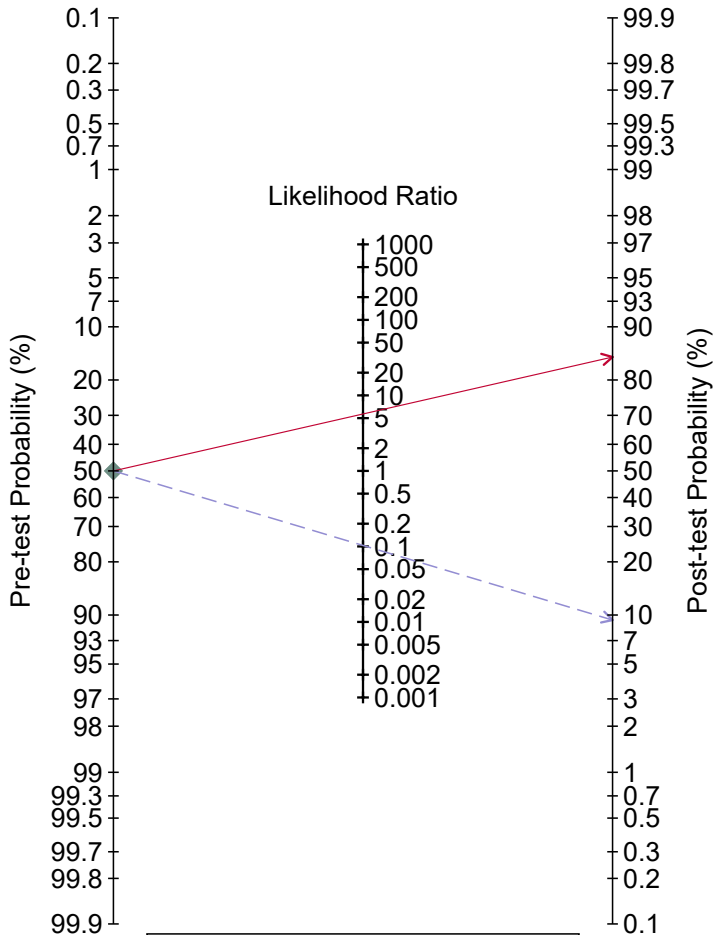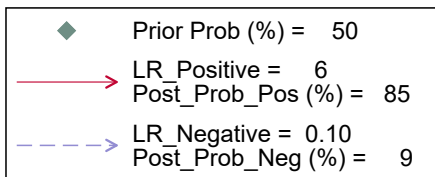

Supplement: Supplementary file 9 — Supplementary Figure 6 [file 12903_2023_3751_MOESM9_ESM.pdf]

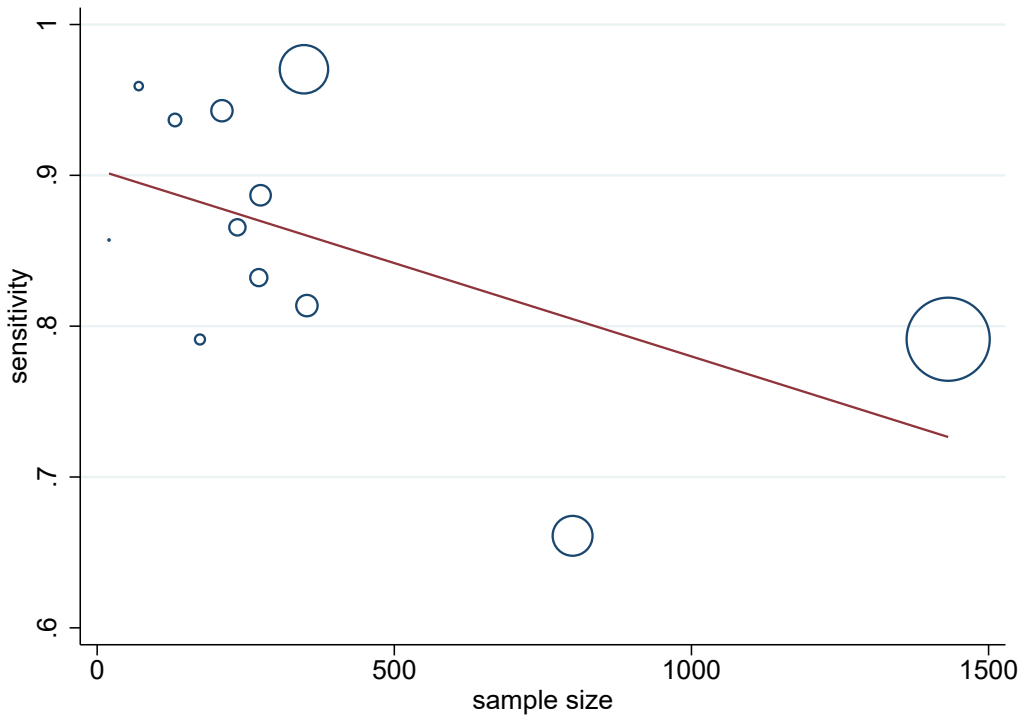

Supplement: Supplementary file 10 — Supplementary Figure 7 [file 12903_2023_3751_MOESM10_ESM.pdf]

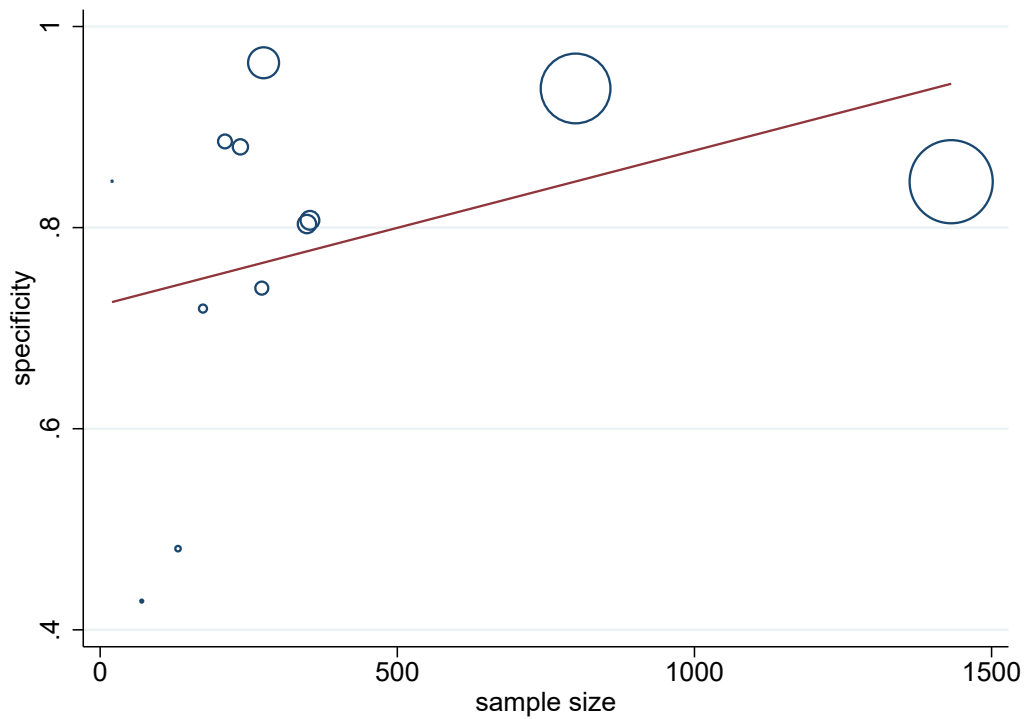

Supplement: Supplementary file 11 — Supplementary Figure 8 [file 12903_2023_3751_MOESM11_ESM.pdf]
